# HSV-1\EGFP stimulates miR-146a expression in a NF-κB-dependent manner in monocytic THP-1 cells

**DOI:** 10.1038/s41598-019-41530-5

**Published:** 2019-03-26

**Authors:** Assunta Venuti, Maria Musarra-Pizzo, Rosamaria Pennisi, Stoyan Tankov, Maria Antonietta Medici, Antonio Mastino, Ana Rebane, Maria Teresa Sciortino

**Affiliations:** 1 Department of Chemical Biological Pharmaceutical and Environmental Sciences, University of Messina, Viale F, Stagno d’Alcontres, 31, Messina, 98166 Italy; 2 Infections and Cancer Biology Group, International Agency for Research on Cancer, 150 Cours Albert Thomas, 69372 Lyon, France; 3 Institute of Biomedicine and Translational Medicine, University of Tartu, Tartu, Estonia; 4 Institute of Translational Pharmacology, CNR, Rome, Italy

## Abstract

The nuclear factor κB (NF-κB) pathway plays a key role in innate and adaptive immunity, cell proliferation and survival, inflammation and tumors development. MiR-146a is an immune system regulator that has anti-inflammatory function in multiple cell types and conditions. Here we demonstrate activation of canonical NF-κB pathway in monocytic cells upon HSV-1 replication. By constructing and using a recombinant HSV-1\EGFP virus, we monitored the capability of the virus to recruit NF-κB and we report that the phosphorylation of p65 protein correlates with an active virus replication at single-cell level. In addition, we found that upregulation of miR-146a during viral replication is strictly dependent on NF-κB activation and correlates with tight control of the interleukin-1 receptor-associate kinase 1 (IRAK1). Accordingly, THP-1 DN IκBα cells, expressing a dominant negative mIκBα, did not show upregulation of miR-146a upon HSV-1 infection. Our data suggest that the expression of miRNA-146a modulates NF-κB activation through targeting IRAK1 during HSV-1 replication in THP-1 cells.

## Introduction

During life cycle, viruses embrace a series of intricate protein-protein interactions with the machineries of the host cell. The qualitative and quantitative characterization of these interactions improves the knowledge on the viral and cellular system. One of the most powerful methods for the analysis uses genetically encoded fluorescent fusion tags for labelling the proteins^1^. In this work, we generated a recombinant HSV-1expressing the *Enhanced Green Fluorescent Protein* (EGFP), named HSV-1\EGFP. The expression of the tagged protein is not affected by viral genes cascade and is maintained constant during all phases of the viral replication. Thus, by using HSV-1\EGFP we explored the capability of the virus to recruit the nuclear transcription factor κB. NF-κB transcription factor plays a major role in the inducible expression of cellular genes involved in the immune, inflammatory and anti-apoptotic responses^2–4^. A wide variety of viruses, belonging to many families, actively manipulates intracellular signaling pathways by inhibiting specific molecular targets in order to elude the immune system^5^. The role of NF-κB in the context of HSV replication has been extensively studied. However, its significance is not fully understood and differences in its regulation seem to depend on specific cellular models. Several studies have demonstrated that HSV-1 activates NF-κB by the interaction between viral structural proteins, such as gD, gH/gL, and UL37, and specific cellular receptors. In particular, we have previously demonstrated that non-replicating wild-type UV-inactivated HSV-1 or purified gD trigger the activation of NF-κB in monocytes following engagement of HSV-1 and/or gD to HVEM receptor^6–11^. Moreover, during viral replication, a second wave of NF-κB activation requires *de novo* HSV-1 genes expression. Indeed, it has been demonstrated that an α gene product, ICP27, is essential to activate NF-κB and UL24 binds the endogenous NF-κB subunits p65 and p50 and reduces the tumour necrosis factor alpha (TNF-α)-mediated nuclear translocation of p65 and p50^12,13^. The activation of NF-κB seems to be important for a productive viral infection by contributing directly to transcriptional regulation of viral genes^14–17^. Diao and collaborators have reported that ICP0 is involved in the NF-κB translocation from cytoplasm to the nucleus^18^. In addition, Amici and collaborators have demonstrated that NF-κB is bound to the ICP0 promoter during viral infection and sustains the ICP0 mRNA transcription^19^. Roberts and collaborators have described that the late protein UL31 is required for an efficient NF-κB activation as well as for an optimal viral protein expression^20^. In different conditions, the NF-κB pathway activation, in response to viral infection, plays an essential role in dsDNA-triggered IFN-β activation and its involvement is critical for HSV-1 replication^21^. Therefore, it has been shown that the HSV-1 ubiquitin-specific protease (UL36USP) inhibits the double-stranded-DNA-mediated NF-κB activation as a mechanism to escape the host antiviral innate immunity^22^. In addition, the HSV-1 DNA polymerase processivity factor UL42 inhibits TNFα-induced NF-κB activation by interaction with p65 and p50 proteins^23^. The above results reveal that there are several layers of recruitment of NF-κB during HSV infection, suggesting that HSV-1 uses the NF-κB factor to improve its replication and controls, through viral proteins expression, the antiviral role of NF-κB signalling also. Recently, in U937 cells has been demonstrated that NF-κB activation simultaneously acts as an antiviral response as well as a mechanism to limit the apoptotic damage in response to HSV-1 infection^24^. However, the molecular mechanisms, downstream to NF-κB activation mediated by HSV-1 infection, are still not fully known in monocytic cells. The canonical NF-κB pathway, triggered by microbial and viral infections, allow to dimers formation containing RelA (also known as p65), c-Rel, or p50 proteins, which are normally retained in the cytoplasm by inhibitors of κB proteins (IκBα, IκBβ, IκBε, IκBγ and Bcl-3). The viral infections can target the β-subunit of Iκ kinases (IKKs) complexes. Iκ kinases (IKKs) phosphorylate IκBs (inhibitors of κB) that bound to NF-κB, resulting in an ubiquitin-dependent degradation of IκBs and translocation of NF-κB dimers to the nucleus^25^. In this study we attempted to identify the components involved in NF-κB signaling cascade in THP-1 monocytic cells by using HSV-1 virus tagged with EGFP used as a reporter gene. In addition, in order to analyze the molecular signals downstream to NF- κB activation, we explored the recruitment of a specific cellular miRNA in the context of HSV-1 infection. Indeed, several investigations have supported the role of miRNAs in physiological functions such as immune response, cell proliferation, cell death and inflammation, which are also known to be regulated by NF-κB. The miR-146a was first identified as an immune system regulator and it has been associated with several diseases such as cancer, viral infections and autoimmune diseases^26^. Particularly, miR-146a has been reported to regulate the NF-κB pathway in response to microbial infections. Taganov and collaborators showed that the expression of miR-146a was increased in THP-1 cells in response to LPS-induced TLR4 activation^27^. During viral infection, the upregulated expression of miR-146a could represent an immunological escape mechanism adopted by viruses to improve their survival into the cell. The vescicular stomatitis virus (VSV) infection, for example, induces the upregulation of miR146a in a RIG-I/NF-κB dependent manner, which inhibits the host antiviral response^28^. An efficient replication of human cytomegalovirus was detected, in MRC-5 cells, by suppressing type I IFN response following host miR-146a upregulation^29^. Other cellular miRNAs are known to be altered by viral infection and at the same time, enhance the viral replication. In particular, miR-23a was found to facilitate HSV-1 replication by targeting the interferon regulatory factor 1 (IRF1) and inhibiting the interferon pathway^30^. Given to the importance of miRNAs functions and their role in the regulation of cellular antiviral signaling, we mainly studied the correlation between HSV-1, NF-κB and miR-146a. We suggested for the first time a negative feedback, in a monocytic cellular model, where the viral infection recruits NF-κB and stimulates the accumulation of miR-146a; this in turn reduces the intracellular levels of IRAK1 shutting down the NF-κB response. Here we report a novel immunological escape mechanism adopted by HSV-1, which involves the activation of a signalling network and allows to improve of viral replication.

## Results

### Growth properties of the HSV-1 expressing EGFP protein (HSV-1\EGFP) mutant virus

**Figure 1:**
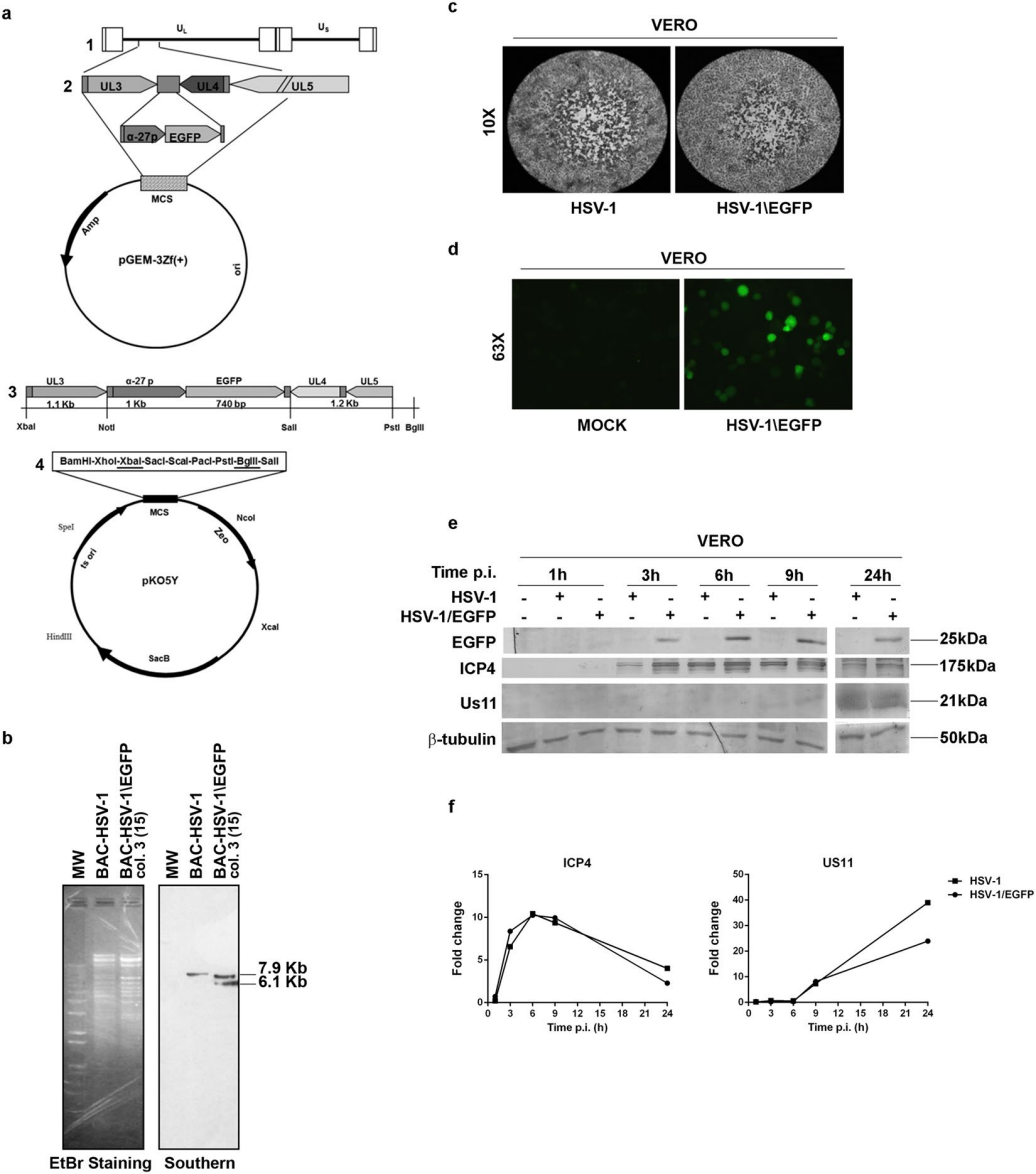
Schematic representation and structure of the recombinant HSV-1\EGFP. (**a**) Schematic representation of plasmid DNAs. Line 1, linear representation of the HSV-1 genome. Rectangles represent the inverted repeats flanking the unique sequences (UL and US, represented by thin lines). Line 2, pGEM-3Zf (+) DNA plasmid containing the insertion of HSV-1 sequence encoding UL3 to UL5. Arrows indicate the polarity and position of each open reading frame. Line 3, an expanded section of the HSV-1 domain plus an additional α27 promoter gene in frame with EGFP sequence inserted between the restriction sites XbaI and PstI. Line 4, XbaI/BglII fragment from the digested pGEM-UL3-α27-EGFP-UL4/UL5 transfer plasmid, resulting in a 4,040-bp fragment, was transferred to shuttle plasmid pKo5Y at the XbaI/ BglII sites. (**b**) Verification of the structure of BAC-EGFP DNA. BAC-HSV-1 and BAC-HSV-1\EGFP DNAs, purified from RR1 bacterial cells, were separately digested with EcoRV. DNA was separated by agarose gel electrophoresis and hybridized with a biotinylated probe that recognizes both wt α27promoter and the α27-promoter controlling early EGFP expression (**c**) Growth properties of wild-type HSV-1 and HSV-1\EGFP mutant virus. Plaque formation in Vero cells infected with wild-type HSV-1 and HSV-1\EGFP virus is shown. Following infection, cells were overlaid with a methylcellulose-containing medium for 3 days. Cells were then fixed, stained with crystal violet, and visualized with an inverted microscope (higher magnification, ×10); (**d**) expression of EGFP in Vero cells infected with the HSV-1\EGFP. Confluent cell monolayers were uninfected (mock) or infected with HSV-1\EGFP, collected 24 h p.i. and visualized by fluorescence microscope (Leitz, Wetzlar, Germany); (**e**) Vero cells were mock infected or infected with HSV-1 and HSV-1\EGFP at MOI 10, separately, and collected at 1, 3, 6, 9 and 24 hrs. Equal amounts of proteins were processed as described in Materials and Methods to analyse the expression of α (ICP4), γ2 (US11) and EGFP proteins. An anti-β-tubulin antibody was used as a control. The grouping blots are cropped from two different gels, as displayed in the figure with the white space. (**f**) Band density was determined with the T.I.N.A. program, and was expressed as fold change over the appropriate housekeeping genes.

Several groups have cloned HSV-1 genome into a F plasmid named BAC (Bacterial artificial chromosome); by so doing, it is possible to stably maintain the viral genome as BAC in *Escherichia coli* and to mutagenize the viral genome in bacterial cells by using their recombination machinery. Indeed, the reconstitution of viral particles is obtained after transfection of the recombinant BAC plasmid in mammalian cells^31–33^. To explore the capability of HSV-1 to recruit NF-κB, we generated a recombinant virus expressing EGFP under the control of α27 promoter, which can control the expression of the protein since the early phase of the replication in concurrence with the alpha class of HSV-genes, named HSV-1\EGFP. The mutant HSV-1 virus was generated by using the HSV-1 (F) bacterial artificial chromosome (BAC-HSV-1) with the transfer plasmid pKo5Y (pRB5708), as schematically illustrated in Fig. 1a and described in detail in Materials and Methods. The sequence of EGFP was inserted in the intergenic region between the UL3 and UL4 genes without deletion of any viral sequence; of note, it has been reported that the insertion of exogenous genes into this site is stable and had no effect on viral growth in cell culture^34,35^. For the isolation of recombinant clone in bacterial cells, we screened the viral DNA for the presence of the EGFP sequence by PCR (data not shown), and BAC-HSV-1\EGFP was analyzed in comparison with BAC-HSV-1 by Southern blotting (Fig. 1b). Equal amounts of BAC-HSV-1 and BAC-HSV-1\EGFP plasmid DNAs extracted from RR1 bacteria were digested with EcoRV, electrophoretically separated on an agarose gel, and hybridized with a biotinylated DNA probe containing the sequence of α27 promoter. Considering the construction of the virus, the probe hybridized with a 7.9-kb fragment only in HSV-1 wt DNA and with two fragments, 7.9-kb and 6.1-kb, in HSV-1\EGFP DNA. This strategy allowed us to recognize both the wt α27-promoter, which controls the expression of ICP27 gene and the α27-promoter which controls early expression of EGFP (Fig. 1b). These results indicated that BAC vector harbors the EGFP sequence inserted into the intergenic region of the UL3 and UL4 genes, under control of α27 promoter. Next, we examined the growth properties of the HSV-1\EGFP. In deep, Vero cells were exposed to 1 PFU/cell of HSV-1 and HSV-1\EGFP respectively; at 24 h after infection, the cells were harvested and the virus yield was titered on Vero cells (data not shown). In addition, the plaque size-morphology obtained for HSV-1\EGFP was comparable with the wild-type ones (Fig. 1c). We also evaluated the capability of the recombinant virus to express the EGFP tag by fluorescence analysis. Confluent cell monolayers were infected with HSV-1\EGFP recombinant virus or not as described in Materials and Methods. The fluorescence imaging clearly demonstrated the capability of the recombinant HSV-1\EGFP virus to express EGFP protein (Fig. 1d). The characterization of the recombinant virus was implemented by analyzing the protein expression of the α gene ICP4 and the γ^2^ late gene Us11, as representative proteins of the viral gene cascade^36,37^ and the expression of the EGFP reporter gene. Vero cells were infected with HSV-1 and HSV-1/EGFP at MOI 10, separately, and collected at 1, 3, 6, 9, 24 hrs p.i. for western blot analysis. The expression of the EGFP protein was detectable starting at 3 h p.i. and it remained stable until the late stage of viral replication (24 h) as shown in Fig. 1e. In addition, the EGFP integrated into HSV-1 genome has no impact onto ICP4 and Us11 protein expression (Fig. 1e,f). Indeed, the trend of the protein expression observed with the recombinant HSV-1\EGFP virus in fully permissive cells was comparable to that observed with the wild type HSV-1 (wt HSV-1) (Fig. 1f). Taken together, these data suggest that the recombinant virus has the same biological properties as the wt HSV-1, thus we were able to use the recombinant virus in the next experiments.

### HSV-1\EGFP is able to replicate in THP-1 cells

It is well known that HSV-1 infects monocytic cells, such as human acute monocytic leukemia cells (THP-1), but the degree of permissiveness is reduced compared to Vero cells, for this reason they are considered a system “not fully permissive”^38^. Particularly, HSV-1 is not fully competent to replicate in non-activated THP-1 cells at low MOI. Thus, we performed a time course analysis to characterize the capability of HSV-1\EGFP to replicate in THP-1 cells by infecting cells at MOI 50 PFU/cell based on our previous study^6^. It has been reported that no necrosis was found at 24 and 48 h p.i., as a consequence apoptotic cell death was evaluated in THP-1 cells exposed to HSV-1 and HSV-1\EGFP at MOI 50 during late time points. The data showed that at 24 h and 48 h p.i. the percentage of apoptotic cells, detected by Acridine Orange staining, was 15% and 20% respectively, indicating that the majority of cell population was still alive. Only at 72 h p.i. we had a high % of apoptotic cell death due to active replication of the virus (See Supplementary Fig. S1).

**Figure 2:**
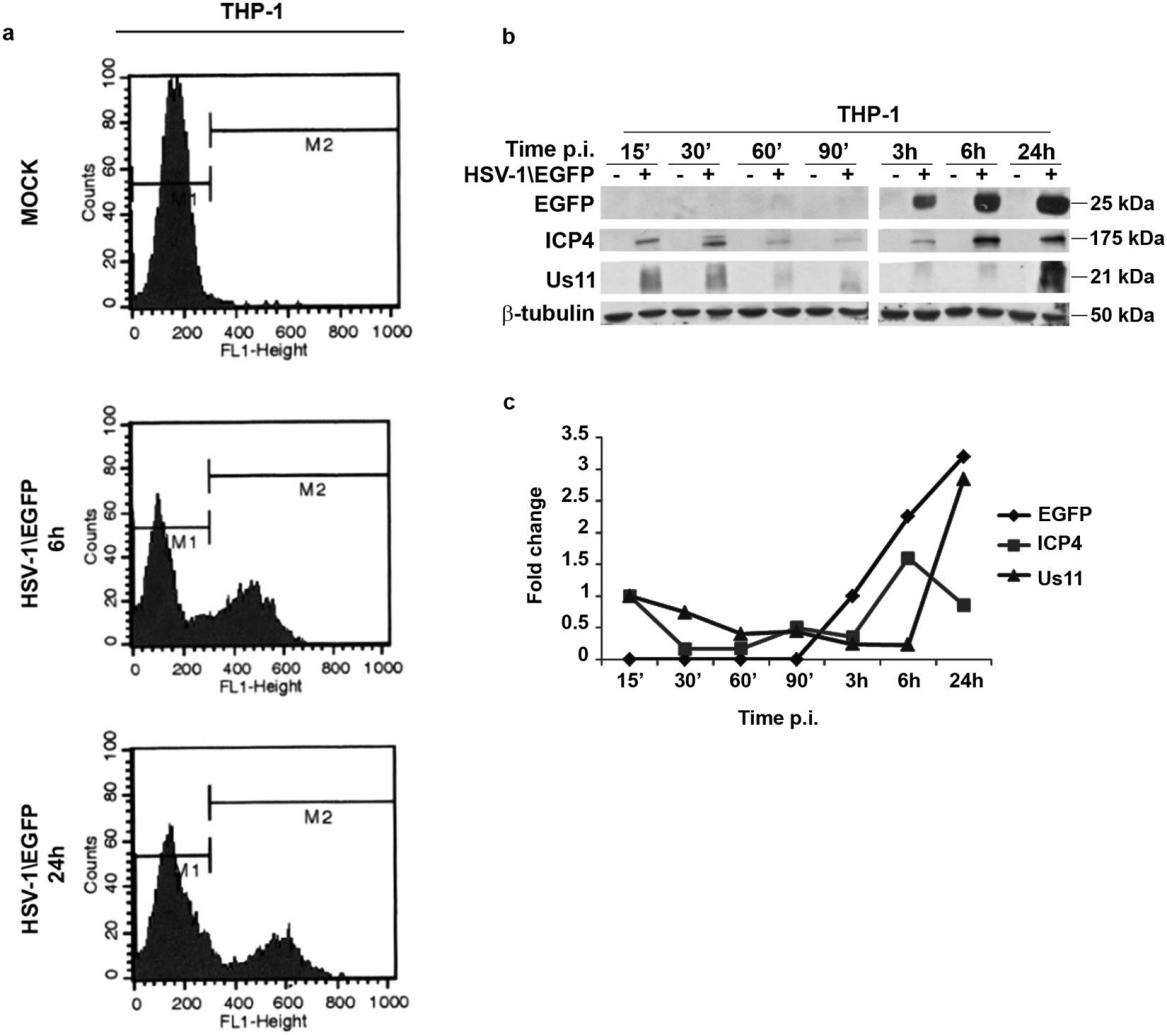
Characterization of the recombinant HSV-1\EGFP. (**a**) EGFP autofluorescence of recombinant HSV-1\EGFP was determined by FACS analysis as described in Materials and Methods. THP-1 wild type cells were mock infected or infected with HSV-1\EGFP at MOI 50 and collected at 6 and 24 hrs p.i. Flow cytometry analysis allowed us to select cell showing high levels of auto-fluorescent EGFP. (**b**) wt THP-1 cells were mock infected or infected with HSV-1\EGFP at MOI 50 and collected at different times p.i. (15′, 30′, 60′, 90′, 3, 6, and 24 hrs). The cells were harvested at the indicated times and equal amounts of proteins were processed as described in Materials and Methods. After transferring onto a nitrocellulose sheet, were reacted with antibodies against representative α (ICP4), γ_2_ (U_S_11) and EGFP proteins. An anti-β-tubulin antibody was used as a control. The grouping blots are cropped from two different gels, as displayed in the figure with the white space. (**c**) Band density was determined with the T.I.N.A. program, and was expressed as fold change over the appropriate housekeeping genes.

In addition and accordingly with the data obtained in Vero cells, the FACS analysis showed that the recombinant virus is able to infect the cells by expressing EGFP protein both at 6 h and 24 h post infection, with 36.95% and 26.30% of EGFP positive cells respectively (Fig. 2a). To complement this finding, we analyzed the accumulation of proteins belonging to different kinetic classes. The cells were exposed or not to 50 PFU/cell of HSV-1\EGFP and then collected at 15′, 30′, 60′, 90′, 3, 6, 24 hrs after infection. The results, obtained by western blot, shown in Fig. 2b,c, suggested the following: (i) the expression of EGFP reporter gene was detected starting at 3 h post infection with a progressive and relevant increase until 24 h; (ii) the viral α gene ICP4 was detectable already at 15′ p.i., probably derived from parental virions, with a subsequent neo synthesis starting at 3 h, a peak of expression at 6 h and a final decrease in the late stage of viral replication; (iii) based on viral genes cascades, the accumulation of γ^2^ Us11 as a late proteins, increased at late time 24 h during viral replication. These data taken together suggest the possibility to use the recombinant HSV-1\EGFP virus as an experimental model to explore the capability of the virus to recruit the NF-κB.

### Exposure to HSV-1 induces the activation of NF-κB in THP-1 actively expressing EGFP tag

**Figure 3:**
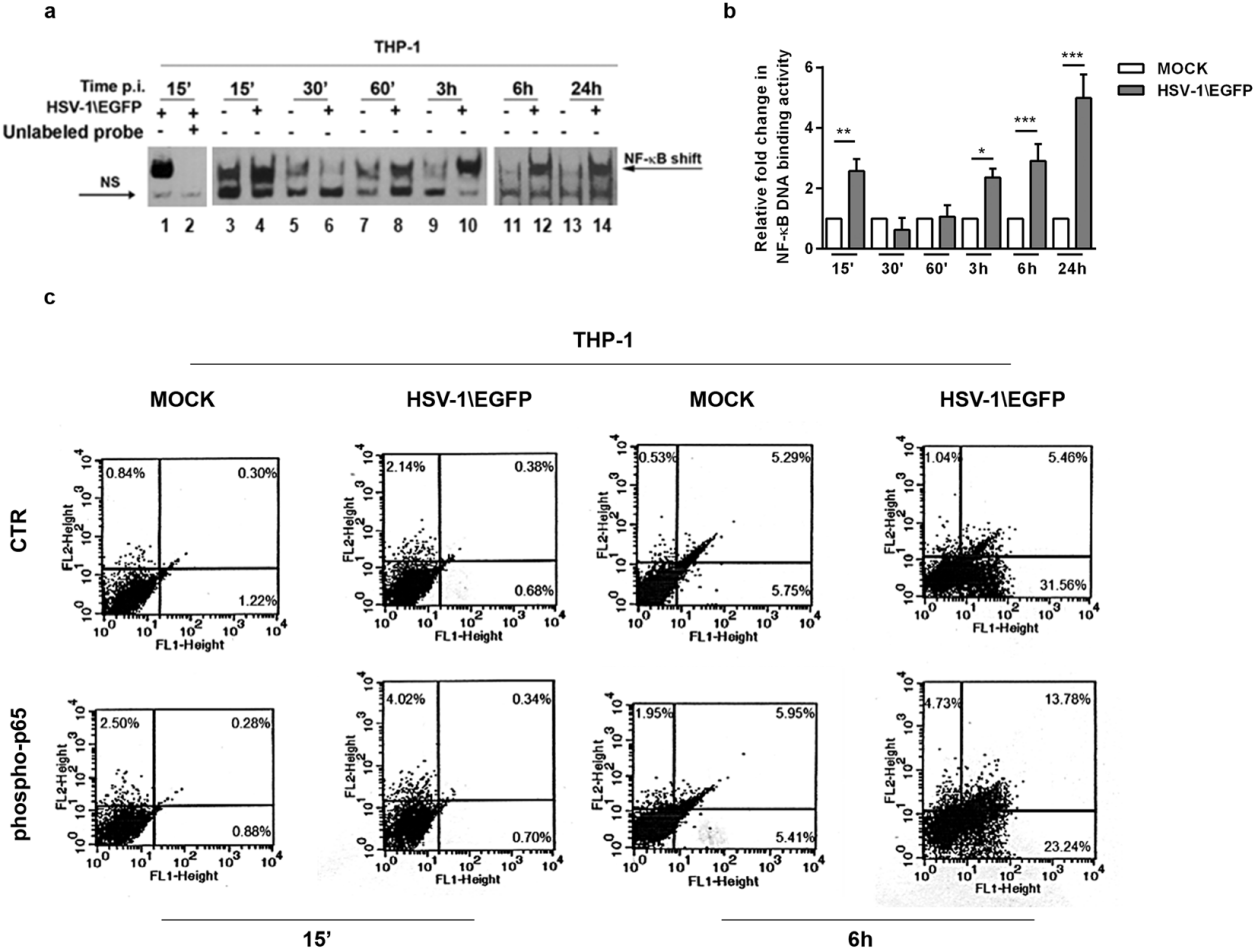
Analysis of activation of NF-κB by EMSA in THP-1 monocytic cells infected with recombinant HSV-1\EGFP. (**a**) wt THP-1 cells were mock infected or infected with HSV-1\EGFP at 50 MOI. A) 15′, 30′, 60′, 3, 6, and 24 hrs after exposure to virus, samples were collected and evaluated by EMSA technique (lanes 3–14). The position of the NF-κB DNA and NS are indicated; for the competition assay unlabelled kB DNA probe (agttgaggggactttcccaggc) was used to test the specificity of the analysis (lane 2 versus lane 1). The grouping gel shift were cropped from two different gels, as displayed in the figure with the white space. (**b**) quantitative analysis using densitometry image of the EMSA was determined with the T.I.N.A. program, and was expressed as fold change over the non specific band (NS). (**c**) wt THP-1 cells were mock infected or infected with HSV-1\EGFP at MOI 50 and collected at 15′ and 6 hrs p.i. Flow cytometry analysis allowed us to select cell showing high levels of auto-fluorescent EGFP and the reactivity with anti-human phospho-p65 antibodies following cell staining.

We have previously demonstrated that THP-1 cells are sensitive to the activation of NF-κB by exposure to UV-inactivated HSV-1 where the virus was unable to replicate but still capable to trigger NF-κB activation by binding its natural receptor^8^. Here, for the first time we show that HSV-1 is able to activate the transcription factor NF-κB during HSV-1\EGFP replication in a time-dependent manner in THP-1 cellular system. The cells were infected or mock infected with HSV-1\EGFP at MOI 50 and kinetically collected at 15′, 30′, 60′, 3 h, 6 h and 24 h p.i. The nuclear extracts were prepared and DNA-binding activity of NF-κB was detected by non-radioactive electrophoretic mobility-shift assay (EMSA). As shown in Fig. 3a, an increased NF-κB activation occurred in cells exposed to HSV-1 “in a two-waves manner” compared to mock-treated control cells, as quantified by densitometric analysis (Fig. 3b). The early activation of NF-κB is dependent on the virus binding to the cell receptors as reported in Sciortino *et al*. 2008^8^, whereas activation of NF-κB at late stage of virus replication correlates to *de novo* viral protein synthesis. Considering that THP-1 cells are not fully permissive to HSV-1 replication, we wanted to investigate the correlation between NF-κB activation and the expression of the EGFP tag protein of recombinant HSV-1\EGFP virus, which indicates the index of active replication. In our previous publication, we showed that exposure of THP-1 cells to UV-inactivated HSV-1 results in enhanced levels of binding activity of NF-κB. The addition of antibodies specific to p50 and to p65 subunits to the nuclear extracts gave rise to the supershift of detectable bands in EMSA clearly upregulated by UV-inactivated HSV-1^8^. Based on the above data, we measured the phosphorylation status of one of NF-κB active subunit, phospho-p65 protein, in the presence or not of recombinant HSV-1\EGFP virus. Flow cytometry analysis allowed us to select cell expressing phospho-p65 protein and showing high levels of autofluorescent EGFP protein following cell staining. As shown in Fig. 3c, we report that the expression of the reporter gene EGFP correlates with the expression of phospho-p65 protein by two-fluorescence. The results indicated that human phosphorylated-p65 protein was detectable in cells expressing high levels of the EGFP tag (Fig. 3c). Definitely, we show that at 6 h after infection, an amount of THP-1 cells expressed phospho-p65 and around 15% of these cells fall in the double-positive quadrant of the cytogram, thus showing that they were cells in which HSV-1 was actively replicating. In conclusion, these results demonstrate that phospho-p65 protein expression correlates with an active virus replication at single-cell level.

### Canonical activation of NF-κB in THP-1 infected cells is mediated by active HSV-1 replication

**Figure 4:**
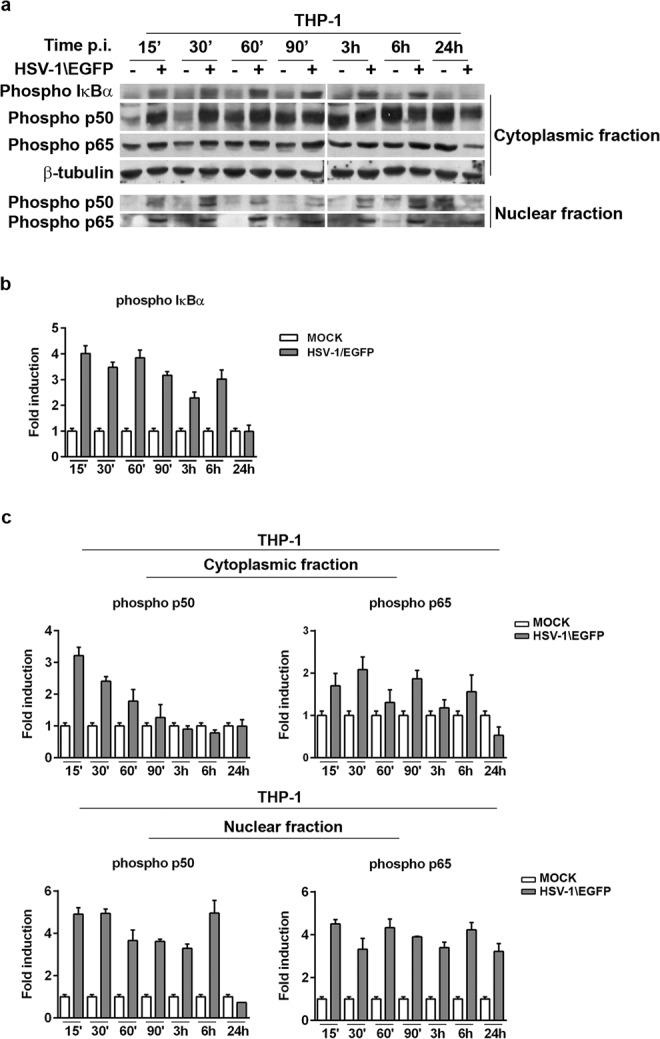
Expression of phospho-p50/p65 proteins in THP-1 cell lines infected with HSV-1\EGFP. (**a**) Wild type THP-1 cells were infected or mock infected with HSV-1\EGFP at MOI 50 and collected at different times p.i. (15′, 30′, 60′, 90′, 3, 6, and 24 hrs). Equal amounts of cytoplasmic and nuclear proteins, based on protein quantification (Material and Methods), were separated by polyacrylamide gel electrophoresis and probed with phospho-p105, and -p65 antibodies. In THP-1 wt the phospho-IκBα was evaluated. β-tubulin was used as housekeeping for the cytoplasmic fractions. For the nuclear fractions, the fold change of the infected cells was calculated with the respect to the relative uninfected controls. The grouping blots are cropped from two different gels, as displayed in the figure with the white space. (**b**,**c**) Band density was determined with the T.I.N.A. program, and was expressed as fold change over the appropriate housekeeping gene.

As reported above, the kinetic during the first 24 hours of HSV infection in THP-1 cells shows the activation of the NF-κB complex mainly localized at nuclear level. This is in accordance with the EMSA data analysis. Inducing stimuli leading to phosphorylation and degradation of the inhibitor IκB proteins, is a key event involved in the release of the NF-κB complex. Thus, we wanted to verify first the phosphorylation status of the IκBα that in turn induces the phosphorylation with consequent migration from cytoplasm into the nucleus of NF-κB complex by means p50/p65 complex. As expected, the kinetic showed the hyperphosphorylation status of IκBα at all-time assayed, indicating the potential involvement of the canonical pathway in the activation of NF-κB in THP-1 cells during HSV-1 infection (Fig. 4a,b). Based on these findings and on literature data^13^, next we investigated, in deep detail, the signaling related to the activation of NF-κB during viral replication by measuring the phosphorylated NF-κB complexes by means p50/p65. While nuclear translocation is the primary method of regulating NF-κB activity, we monitored the migration from the cytoplasms into the nuclei of phosphorylated p50/p65 protein complex. At all-time considered, the data definite the accumulation of phosphorylated p50/p65 complex in cells infected with HSV-1\EGFP in comparison with the non-infected one in both cytoplasmic and nuclear fractions, with the exception of p50 protein, which demonstrated a reduction of phosphorylated form at 24 h p.i. The data confirm our previous observations obtained by EMSA analysis (Fig. 4a,c). In conclusion, analysis of IκBα protein expression in THP-1 cells infected with HSV-1\EGFP showed an early and bimodal kinetic, with consequent recruitment of p50/p65 proteins which is coherent with a canonical activation of NF-κB pathway.

### MiR-146a is a responsive gene induced by HSV-1 in a NF-κB-dependent manner

**Figure 5:**
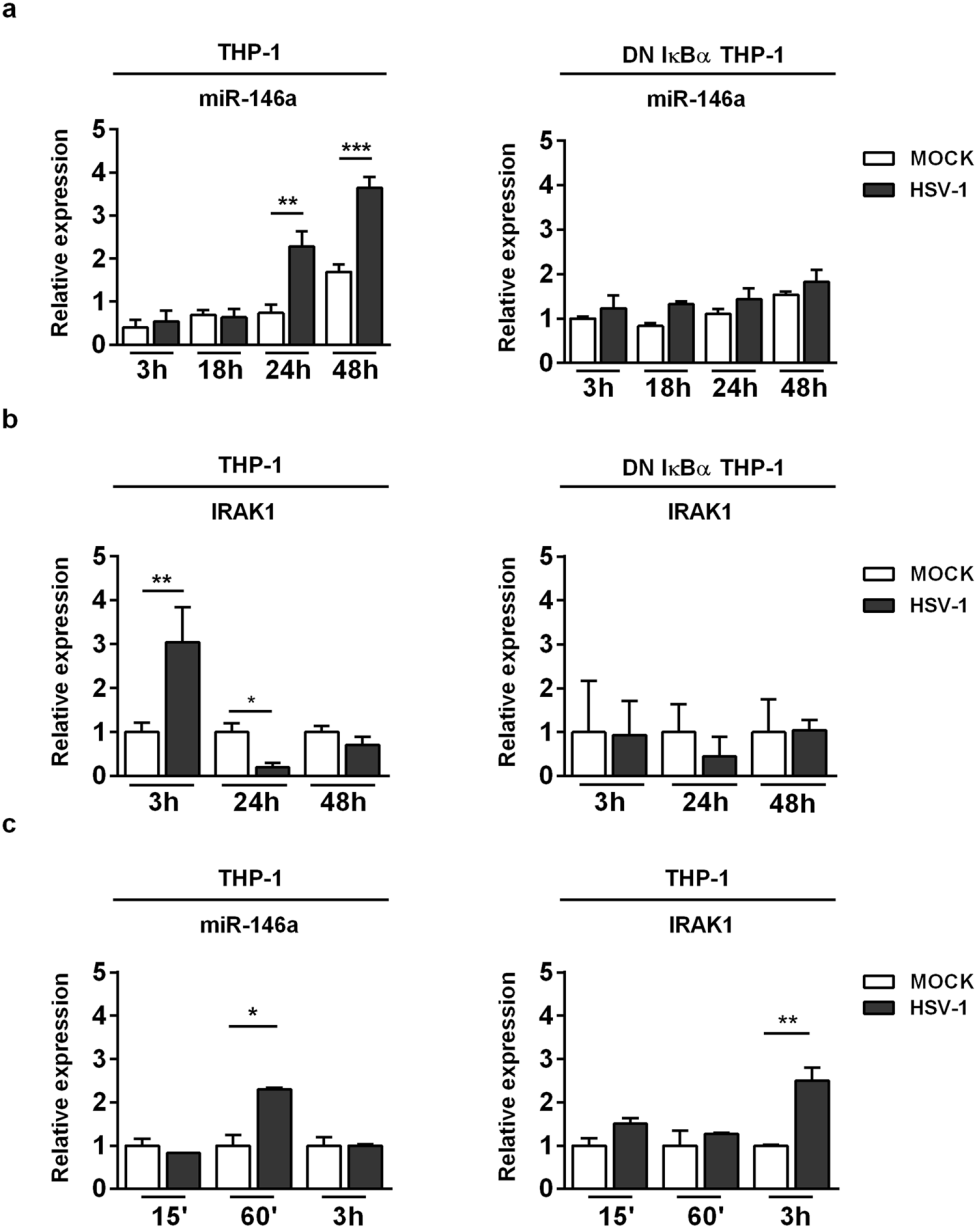
NF-κB-dependent activation of miR-146a in HSV-1-infected monocytic leukemia cell line. (**a**) THP-1 and DN IκBα THP-1 cells were infected with HSV-1 at MOI 50 and collected at the indicated time p.i. MiR-146a expression level in HSV-1 infected THP-1 cells compared to DN IκBα THP-1 infected cells; (**b**) Analysis of miR-146a-target IRAK1 expression level in THP-1 and DN IκBα THP-1 cells infected or mock infected with HSV-1. (**c**) Expression pattern of miR-146a and IRAK1in THP-1 cells at early stages of infection. THP-1 cells were mock infected or infected with HSV-1 at MOI 50 PFU/cell and collected at 15′, 60′ and 3 h p.i. and qPCR analysis were performed. Mean ± standard error of the mean (SEM) is indicated. *p < 0.05; **p < 0.01; and ***p < 0.001.

MiR-146a is expressed widely throughout the hematopoietic system, and generally, its expression increases with maturation and activation of the cell system. Based on this, the expression pattern of microRNAs differs considering the cell types used. Moreover, recent studies have indicated that miR-146a is involved in virus-host cell interaction. Particularly, viral infection can stimulate miR-146a expression in a NF-κB-dependent manner^28^. Based on these published data, we investigated the miR146a expression levels in THP-1 and DN-IκBα THP-1 cell lines following HSV-1 infection. THP-1 and DN-IκBα THP-1 cells were infected or not with HSV-1 at MOI 50 PFU/cell and collected at 3, 18, 24 and 48 hrs p.i. The significant induction of miR-146a upon HSV-1 infection occurred in wild type THP-1 cells (wt THP-1) only and not in DN-IκBα THP-1 cells; as shown by qPCR analysis its expression reached a peak at 48h post infection indicating a specific response related to HSV-1 replication (Fig. 5a). In addition, we also found that the expression pattern of miR-146a differed considering the cellular model used. Indeed, to implement our finding we performed a qPCR analysis of miR-146a in HEp-2 cells, a non-immune cell system, permissive to HSV-1 replication. HEp-2 cells were mock infected or infected with HSV-1 at MOI 10 and collected at 24 h and 48 h p.i. to perform qPCR analysis. Interestingly, miR-146a was undetected in both infected and uninfected HEp-2 cells, but THP-1 infected cells only, considered as a positive control, showed a transcriptional level on miR-146a (See Supplementary Fig. S2). Next, we investigated whether miR-146a expression, mediated by HSV-1 in THP-1 cells, modulates downstream target genes involved in cellular innate response to viral infection. As known, miR-146a directly target several serine/threonine kinases, such as interleukin-1 receptor-associated kinase 1 (IRAK1)^27,39^. According to these data, we analyzed the expression at transcriptional level of IRAK1 in THP-1 and DN-IκBα THP-1 cells infected with HSV-1 at indicated time post infection. As shown in Fig. 5b, a relevant increase in mRNA level of IRAK1 was observed at 3 h p.i. in THP-1 infected cells only, compared to uninfected cells. It was negatively regulated in the late phase of HSV-1 replication and correlated with high expression of miR-146a shown in Fig. 5a. On the contrary, DN-IκBα THP-1 did not show a significant modulation of IRAK1 mRNA level mediated by HSV-1 (Fig. 5b). To note, mRNA level of IRAK1 significantly decreased during HSV-1 infection in THP-1, probably depending on related increase of miR146a level at late stage of infection. To investigate whether the down regulation of IRAK1 lead to the repression of NF-κB signalling, we also analyzed the transcriptional level of p50 and p65 at 24 h p.i. in THP-1. The data indicating that neither activation nor deregulation was found in cells infected with HSV-1 compared to uninfected control cells (See Supplementary Fig. S3). These data demonstrated that the down regulation of IRAK1 mediated by overexpression of miR146a (Fig. 5a,b), clearly lead to a negative regulation of NF-kB signalling pathway at later time point. In addition, we analyzed the expression levels of miR-146a and its target IRAK1 in THP-1 during the early stages of viral replication in order to investigate the expression pattern at earlier time points. THP-1 cells were mock infected or infected with HSV-1 at MOI 50 PFU/cell and collected at 15′, 60′ and 3 h p.i. and qPCR analyses were performed. Interestingly, we found that miR-146a was upregulated at 60′ p.i. in THP-1 infected cells compared to mock cells (Fig. 5c), as a result of NF-κB activation following the virus attachment to the cell receptors. However, transcriptional levels of IRAK1 increased at 3 h p.i. only, in THP-1 infected cells compared to uninfected cells as shown in Fig. 5c. These data indicate that the early activation of miR146a (Fig. 5c) does not lead to down regulation of IRAK1 as we observed during later time points (Fig. 5b). This can be explained considering that the upregulation of IRAK1 at early time point may also be dependent on other cellular and viral factors and may exert different functions. On the contrary, the overexpression of miR146a at later time point (Fig. 5a,b) clearly lead to the down regulation of IRAK1, which results in a negative regulation feedback of NF-kB signalling at transcriptional level. Definitely, we demonstrate that miR-146a might be an HSV-1 primary-responsive gene sensitive to NF-κB activation. In addition, according to previous data, our findings suggest that expression of NF-κB-sensitive miR-146a could be a candidate for the modulation of NF-κB activation through targeting adaptor protein IRAK-1 during HSV-1 replication.

### Overexpression of miR146a negatively regulates IRAK1 in THP-1 infected cells

**Figure 6:**
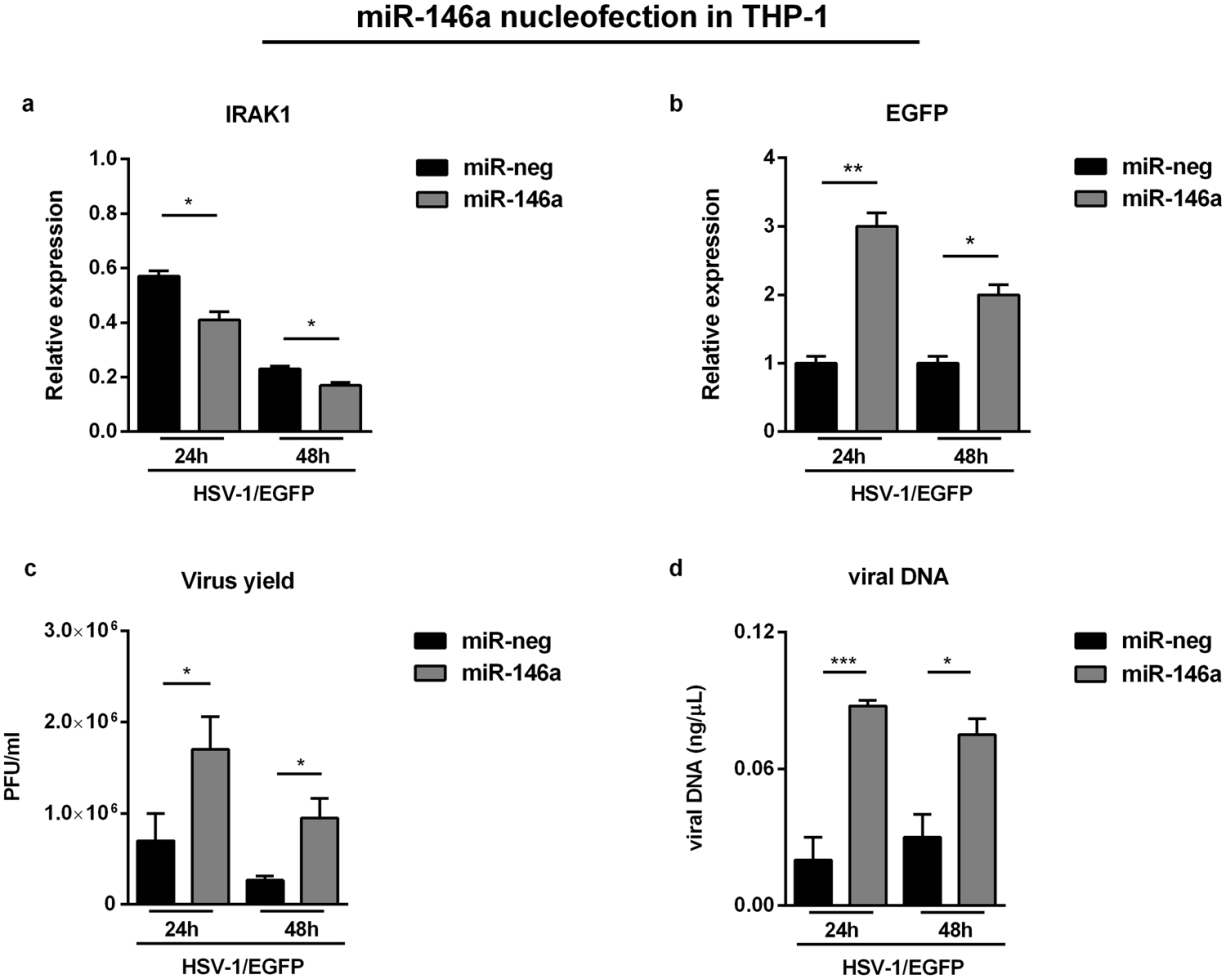
Role of miR-146a in the biology of HSV-1. THP-1 cells were nucleofected with 300 nM of miR-146a mimic or miRNA mimic negative control, followed by infection with HSV-1\EGFP at MOI 50. The cells were collected at 24 h and 48 h p.i. to perform qPCR analysis and titration. (**a**,**b**) mRNA expression levels of IRAK1 and EGFP; (**c**) Virus yield quantified by standard plaque forming assay; (**d**) viral DNA quantified from Ct using the standard curve method and expressed as concentration of ng of DNA for µl. Mean ± standard error of the mean (SEM) is indicated. *p < 0.05; **p < 0.01; and ***p < 0.001.

Recent data demonstrated that host miR-146a promotes replication of human cytomegalovirus by suppressing type I IFN response in MRC-5 cells^29^. Based on this, in order to investigate whether miR-146a can affect HSV-1 replication, we performed nucleofection experiment to overexpress exogenous miR-146a in THP-1 cells, followed by infection according to experimental procedure. Briefly, 300 nM of miR-146a mimic or miRNA mimic negative control were used to nucleofect 2 × 10^6^ cells. The cells were then seeded onto 6 well plates for 6 hours, then infected or not with HSV-1\EGFP at MOI 50 PFU/cell and collected at 24 h and 48 h p.i. for: (i) analysis of IRAK1 and EGFP mRNA levels; (ii) quantization of viral DNA; (iii) evaluation of the virus yield by plaque assay. The overexpression of miR-146a resulted in a significant decrease of transcription level of previously validated direct target IRAK1 in HSV-1 infected cells compared to the control cells treated with the miRNA negative control, indicating that miR-146a inhibits IRAK1 expression during HSV-1 infection (Fig. 6a). Conversely, the overexpression of miR146a resulted in a significant increase of transcription levels of EGFP (Fig. 6b). In addition, the amount of infectious virus production increased significantly in THP-1 cells nucleofected with miR146a mimic (Fig. 6c). At the same time, miR-146a overexpression was found to promote the accumulation of viral DNA as shown in Fig. 6d. Taken together, our data demonstrate that the suppression of the NF-κB pathway mediated by miR-146a, through targeting IRAK1, can enhance HSV replication and its survival into the host cells.

## Discussion

Here, we report a novel feedback between NF-κB signalling and miR-146a expression in human monocytic cells infected by HSV-1. NF-κB is one of the major players in the innate immune responses capable to induce antiviral genes, such as interferon (IFN) and IFN-stimulated genes (ISG). Not surprisingly, viruses modulate, positively or negatively, the NF-κB pathway to evade antiviral responses or to promote viral infection. Indeed, it has been deeply reviewed that viruses, belonging to several families, interfere with NF-κB activity at every step of its signalling pathway. For example, Hepatitis C Virus (HCV)^40^, Enteroviruses^41^, HSV-1^42^ and Measles virus^43^ produce viral proteins able to attenuate the antiviral response by inactivating the signaling mediated by PRR receptors or adaptors, upstream of the NF-κB activation. The protease 3 C of Hepatitis A virus^44^ and the polymerase of Hepatitis B Virus^45^ hamper the activation of the trimeric IκB kinase (IKK) and compromise the NF-κB cascade. Epstein-Barr virus (EBV)^46^, HSV-1^47,48^, Poxvirus^49^ and Vaccinia virus^50^ inhibit, directly or indirectly, the nuclear translocation of p65 which represents a crucial step to sustain the antiviral responseNF-κB-mediated. Therefore, during the host-virus co-evolution, viruses have developed multiple tactics to block the NF-κB activity and promote viral survival. Contrariwise, different virus families induce a persistent activation of NF-κB signaling pathway tightly connected to oncogenic transformation. The primary oncogenic viral mediators activate the NF-κB signaling pathway to improve the viral infection fitness. In particular, it has been shown that the binding between gp350/220 glycoprotein of EBV and CD21 cell surface receptor, triggers NF-κB activation, which results in downstream upregulation of CD21 expression^51^. This feedback positive loop increases the cell susceptibility to viral entry. A similar effect is mediated by Tax protein of human T-cell leukemia virus type 1 (HTLV1) and the vFLIP protein of Kaposi sarcoma-associated herpesvirus (KSHV), which, directly or indirectly, activates IKK complex and as a consequence NF-κB to improve oncogenic transformation^52,53^. Therefore, the viral immunopathogenesis is a multistage and complex process that involves a balance between the activation and inhibition of NF-κB. During the last years, it has been reported that small non-coding RNAs, such as miRNAs, act as posttranscriptional regulators of gene expression and, at the same time, modulate or are modulated by NF-κB^54^. Their role in different physiological functions such as immune responses, cell proliferation, cell death, and inflammation allow us to explore in a particular human monocytic cellular model, such as THP-1, the regulatory interplay between miR-146a and NF-κB during HSV-1 infection. Here, we showed that HSV-1 activates NF-κB in THP-1 cells by triggering the upregulation of miR-146a, which down regulates IRAK1 response to increase viral replication. The capability to develop a new recombinant tagged HSV-1 has expanded our toolbox and has provided new opportunities for studies on virus/host interaction at single cell levels^1,55^. The recombinant tagged HSV-1\EGFP virus allowed us to clearly show that the cell population where the virus is actively replicating displays high levels of phopho-p65 (Fig. 3c) as a result of later temporal wave in the NF-κB activation triggered by HSV-1 infection. The early activation of NF-κB, verified at 15 minutes post infection (Fig. 3a), is dependent on the virus binding to the cell receptors^8^, the second wave of NF-κB activation is correlated to *de novo* viral proteins synthesis starting at 3 hours post infection. In both cases, the nuclear accumulation of p65/p50 complex is guaranteed by upstream phosphorylation of IκBα (Fig. 4a,b) consistently with a canonical activation of NF-κB pathway. The discovery of epigenetics and non-coding RNAs has rapidly established that miRNAs are integral components of the downstream NF-κB signalling cascade^56^. Recent findings on the role of miRNAs in the NF-κB signalling cascade indicated that miR-146a is a critical component for regulating the immune response^57^. Here, we showed that the late, rather than early activation of NF-κB, stimulates transcriptional levels of miR-146a (Fig. 5a) and shuts down the expression of the putative target, IRAK1 (Fig. 5b). The broken signalling pathway promotes the accumulation of viral DNA and the production of infective particles (Fig. 6c,d). Our study provides new insights into potential anti-HSV-1 therapy by showing that miR-146a acts as a negative regulator of NF-κB pathway through suppression of IRAK1 as an additional mechanism enrolled by the virus to promote its survival in host cells. As known, an inappropriate activation of inflammation is harmful to an organism and can lead to immunopatological conditions or immune disorders. Recently, it has been demonstrated that knocking out miR-146a or neutralizing virus-induced miR-146a by specific antagomiR promotes IFNβ production by reestablishing the expressions of IRAK1 and TRAF6 and this results in a decrease in viral propagation in a mouse model^58^. It has been reported also that the suppression of miR-122 by a locked nucleic acid-modified oligonucleotides (LNA), attenuates HCV infection and replication in a chimpanzee model^59^. Therefore, our study underlines the interplayed regulatory mechanism between NF-κB and miR-146a during HSV-1 replication, highlighting the possibility to control the NF-κB response as a potential therapeutic strategy in the inflammatory disorders.

## Materials and Methods

### Cells culture and viruses

Wild type acute monocytic leukemia (THP-1) cells kindly provided by Prof. Bernad Roizman (University of Chicago, USA), were maintained in RPMI-1640 medium (Lonza, Belgium) supplemented with 10% FBS (Euroclone). DN IκBα THP-1 cells, stably transfected with a dominant negative mutant IκBα, were maintained under selection with 400 µg/ml of Geneticin (Gibco). HEp-2 cells (human larynx epidermoid carcinoma cell line) were grown in Dulbecco’s modified Eagle’s medium (Gibco/Invitrogen Corporation, Grand Island, NY) supplemented with 10% of fetal bovine serum, 100 U/ml penicillin and 100 mg/ml streptomycin. Vero cells, originally obtained from the American Type Culture Collection, were propagated in DMEM supplemented with 6% FBS. HSV-1 strain F was kindly provided by Prof. Bernard Roizman. A mutant HSV-1\EGFP was constructed by using BAC-System as following described, and was kindly provided by Prof. Bernard Roizman. For experimental infection HSV-1 and HSV-1\EGFP diluted in RPMI or RPMI alone (mock-infected) at multiplicity of infection (MOI) of 50 PFU/mL and collected at the indicated times post infection (p.i.).

### Standard Plaque Assay on VERO cells

Confluent monolayers of VERO cells were cultured in 24 multiwell plates. The infected samples were frozen and thawed three times. Hundred µl of each dilution of the suspension were used to infect the monolayers. The plates were incubated for 1 h at 37 °C. Then, the viral inoculum was replaced with culture medium containing 0.8% methylcellulose. After 72 h the plated were stained with a crystal violet solution and the plaques were visualized and counted by using an inverted microscope.

### Construction of a mutant HSV-1/EGFP

The HSV-1 mutant virus expressing the enhanced green fluorescence protein (HSV-1\EGFP) was constructed by using the HSV-1 (F) bacterial artificial chromosome (BAC-HSV-1) with the aid of the transfer plasmid pKo5Y. To construct the pGEM-UL3-α27-EGFP-UL4/UL5 transfer plasmid, the HSV-1 flanking sequences UL-3, UL-4 and portions of UL-5 gene was inserted into pGEM-3Zf (+) (Clontech Laboratories, Inc) at two compatible XbaI/PstI restriction sites to yield pGEM-UL3-UL4/UL5. The plasmid pRB5260-EGFP, containing the α27 promoter and EGFP gene cassette, was digested with NotI/SalI restriction enzymes, generating a fragment containing the α27 promoter and EGFP gene. This fragment was inserted into the intergenic region between UL-3 and UL-4 of the pGEM-UL3-UL4/UL5 plasmid. The obtained plasmid was mutated by using a Quickchange site-direct mutagenesis kit (Stratagene, USA) along with the following primers: 5′-GCA-GGC-ATG-CAA-GCT-TGA-GAG-ATC-TAT-AGT-GTC-ACC-TAA-ATA-GC- 3′ and its complementary strand. The oligonucleotides created an underlined restriction site BglII in pGEM-UL3-UL4/UL5 plasmid. The plasmid DNA was cleaved with XbaI/BglII and the resulting fragment, UL3-α27-EGFP-UL4/UL5, was subcloned in the shuttle vector pKo5Y. To generate the mutant virus, competent cells that harbored the BAC-HSV-1 (RR1-HSV-1) were transformed with 0.3 μg of shuttle vector DNA, pKo5Y-UL3-α27-EGFP-UL4/UL5, plated on zeocine (Zeo 20 μg/ml) plus chloramphenicol (Cm 20 μg/ml) plates and incubated overnight at 43 °C. After incubation, 8 colonies were picked, plated on Cm/10% Sucrose (Suc) Lennox Broth (LB) plates, and further incubated at 30 °C overnight. Cm/Suc colonies were streaked on Cm/Suc and Zeo plates, separately, and then incubated at 30 °C overnight and were further screened by PCR under the following conditions: 5 min at 95 °C; then, 35 cycles of 94 °C, 1 min; 60 °C 1 min; 72 °C 45 s. The primers used were: EGFP, forward: 5′-GGA-ATT-CAC-CAT-GGT-GAG-CAA-GGG-CGA-G-3′; e EGFP, reverse: 5′-ACG-CGT-CGA-CGA-GCT-CTA-GGG-CCG-CTT-TAC-TTG-3′. A PCR-confirmed colony was selected and the recombinant BAC-HSV-1\EGFP. BAC HSV-1 DNA and recombinant BAC HSV-1\EGFP DNA were extract from RR1 bacterial cells or RR1-HSV-1\EGFP DNA respectively, using Quiagene Max Kit, according to the manufacturer’s instructions.

### Generation of HSV-1\EGFP mutant virus

Vero cells were transfected with 1.2 μg of the recombinant BAC-HSV-1/EGFP DNA with the aid of Lipofectamine Reagent (Invitrogen). The mixture DNA-Lipofectamine was added to the cells and was incubated at 37 °C for 4 h. The cells were incubated for 3 days at 37 °C and then harvested for characterization of HSV-1\EGFP as described below.

### Characterization of HSV-1\EGFP

Vero cells were infected with HSV-1\EGFP for 1 h at 37 °C; after the incubation time, monolayers were incubated with 0.8% methylcellulose. After 3 days cells were fixed, stained with crystal violet, and visualized for plaque detection. Separately, Vero cells infected with or without HSV-1\EGFP were collected at 24 h, then fixed in 4% paraformaldehyde before addition of 0.1% Triton X-100 in PBS. Samples were the analyzed by using a FITC filter Biomed fluorescence microscope (Leitz, Wetzlar, Germany). For analysis of viral proteins expression Vero cells were mock infected or infected with HSV-1 and HSV-1\EGFP at MOI 10, separately, and collected at 1, 3, 6, 9 and 24 hrs and an equal amounts of proteins were processed as described in Materials and Methods.

### Southern blot analysis of viral DNAs

Equal amount (2.5 μg) of BAC-HSV-1 and BAC-HSV-1\EGFP DNAs, purified from RR1 bacterial cells, were digested with EcoRV enzyme, electrophoretically separated on an agarose gel, and transferred to Zeta-Probe membrane (Biorad). The membrane was incubated for 1 h at 65 °C in a pre-hybridization buffer (Na_2_HPO_4_ 0.5 M; EDTA 0.5 M; SDS 20%). The hybridization procedure was performed with specific nick-translated probe overnight. Probe binding was detected by using “Chemiluminescent Nucleic Acid Detection Module” (Pierce, Rockford, IL). The plasmid pRB5260 containing the α27 promoter CDS was used to generate biotin-16-UTP-labeled (Roche Diagnostic, Germany) probe using a Nick Translation Kit (Roche Diagnostic).

### Construction of stable transfectants THP-1 DN IκBα cells expressing constitutively mutant murine IκBα

Dominant negative murine IκBα-DNA (pcDNA-dn mIκBα)^6^ was used to obtain stable expression of mutant murine IκBα (mIκBα), THP-1 cells. The cells were resuspended in fresh medium without serum and antibiotics. A volume of 12 µl of ${{\rm{Fugene}}6}^{{\rm{TM}}}$ (Roche Applied Science) was added to 188 µl of RPMI and mixed with 3 µg of pcDNA-mIκBα construct. The DNA mixture was incubated at r.t. and then added to the cells. After 72 h, the cells were resuspended in medium containing 400 µg/ml of Geneticin (Gibco).

### Electrophoretic Mobility Shift Assay

Nuclear extracts were prepared from infected cells or mock infected collected at different time after infection. Cells were harvested and processed as described by Sciortino *et al.,* 2008^8^.

### Antibodies

Anti-ICP4 and anti-Us11 monoclonal antibody were a gift from Prof. Bernard Roizman. Anti-phospho-p105 NF-κB (Ser933) (#4884), anti-pospho- IκBα (Ser32) (#9241), anti-pospho-NF-κB p65 (Ser536) (93H1) (#3033) MAbs were purchased from Cell Signaling Technology. Anti-β-tubulin was purchased from ICN Biomedicals (Aurora, OH). Anti-GFP (SC-9996), peroxidase-conjugated anti mouse IgG, peroxidase-conjugated anti-rabbit IgG were purchased from Santa Cruz Biotechnology (Santa Cruz, CA). Protein bands were visualized using Super Signal West Pico as a chemiluminescent substrate (Thermo Scientific, Rockford, IL).

### Protein extractions and immunoblot

The isolation of nuclear and cytoplasmic fractions was performed as reported previously^60^. Proteins derived from nuclear and cytoplasmic fractions were quantified by ‘DC Protein Assay’ (Bio-Rad), and equal amounts were used for western blot analysis to evaluate the accumulation of both viral and cellular proteins. Resolved proteins by SDS-PAGE were transferred to nitrocellulose membranes (Biorad). The membranes were incubated overnight at 4 °C with the appropriate primary antibody and successively probed with secondary antibodies. Quantitative densitometry analysis of immunoblot band intensities was performed by using the TINA software (version 2.10, Raytest, Straubenhardt, Germany).

### FACS analysis

To detect the cellular expression of EGFP, the cells were harvested and were fixed in 4% paraformaldehyde in PBS for 15 min at 37 °C and then analyzed by using a Becton Dickinson FACS analyzer. For the analysis of intranuclear proteins, 2 × 10^6^ cells were fixed in 0.5 ml of 4% paraformaldehyde in PBS for 15 min at 37 °C then, after being washed twice in PBS, in 90% methanol for further 10 min at 4 °C. Sample were first incubated with an antibody against phospho NF-κB p65 (Ser536) for 30 min at room temperature and then stained with a secondary antibody anti-rabbit IgG-PE. The cells were analyzed by using a FACScan flow cytometer and CellQuest software.

### RNA extraction, reverse transcription and real-time PCR analysis

Total RNA was extracted using TRIzol^®^ (Life Technologies) according to the manufacturer’s instructions. Total RNA (1.5 µg) was reverse transcribed using Revert Aid H Minus M-MLV Reverse transcriptase (Thermo Fisher Scientific), under the following conditions; at 42 °C for 60 min, followed by 90 °C for 5 min. The cDNAs were used for quantitative real-time RT-PCR using CepheidSmartCycler II System (Cepheid Europe, France) and Maxima SYBR Green (Thermo Fisher Scientific), under the following conditions: 95 °C for 10 min, 35 cycles at 95 °C for 30 sec/60 °C for 30 sec/ 72 °C for 45 sec. The cDNA copy numbers were normalized to GAPDH. The primers used were: IRAK1 Forw-5′ gctggctactgtgctcagaac, IRAK1 Rev-5′ cagcctctcatccagaaggac; EGFP Forw-5′gagctgaagggcatcgactt, EGFP Rev-5′ ctcaggtagtggttgtcggg; GAPDH Forw-5′ gagaaggctggggctcat, GAPDH Rev-5′ tgctgatgatcttgaggctg. Each quantitative Real-time PCR experiments include a minus-reverse transcriptase control.

### Overexpression of miR-146a in THP-1 cells

Overexpression of miR146a was performed in a 4D-Nucleofector system (Lonza). Briefly, 2 × 10^6^ cells were nucleofected with 300 nM of miR-146a mimic or miRNA mimic negative control according with the manufacturer’s instructions. The cells were then seeded onto 6 well plates for 6 hours, then infected or not with HSV-1\EGFP at MOI 50 PFU/cell and collected at 24 h and 48 h p.i. to perform analysis qPCR analysis and titration.

### miRNA analysis

Total RNA was extracted using TRIzol^®^ (Life Technologies) and purified with the RNAeasy mini kit (Qiagen). Universal reverse transcription (RT) was carried out with TaqMan™ Advanced miRNA cDNA Synthesis Kit (Termo Fisher Scientific) according to the manufacturer’s instructions. TaqMan MicroRNA assays for hsa-miR-146a (478399 mir) and TaqMan Fast Advanced Master mix were used for detection of miR-146a expression level carried out on a ViiA 7 Real-Time PCR System (Thermo Fisher Scientific) under the following conditions: incubation for 20 s at 95 °C, followed by 40 cycles of 3 s at 95 °C and 30 s at 60 °C. Each sample was amplified in triplicate. miR-let7a and miR-361 were used as endogenous control.

### Statistical analysis

Student’s t-test was used for statistical analysis to compare different conditions. Data are expressed as the results of three biologically independent experiments. For the data analysis, the Graphpad Prism 6 software (GraphPad Software, San Diego, CA, USA) was used.

## Supplementary information

Supplementary Data
