# Supplementary material for: HSV-1\EGFP stimulates miR-146a expression in a NF-κB-dependent manner in monocytic THP-1 cells
Source: Sci Rep. 2019 Mar 26;9:5157. doi: 10.1038/s41598-019-41530-5 (PMC6435682; doi:10.1038/s41598-019-41530-5)
Supplement: Supplementary file 1 — Supplementary Data [file 41598_2019_41530_MOESM1_ESM.docx]

**HSV-1\EGFP stimulates miR-146a expression in a NF-κB-dependent manner in monocytic THP-1 cells.**

Assunta Venuti^1,2^***,** Maria Musarra-Pizzo^1^, Rosamaria Pennisi^1^, Stoyan Tankov^3^, Maria Antonietta Medici^1^, Antonio Mastino^1,4^, Ana Rebane^3^, Maria Teresa Sciortino^1^*****.

**Affiliations:**

^1^Department of Chemical Biological Pharmaceutical and Environmental Sciences, University of Messina, Viale F. Stagno d’Alcontres, 31, Messina, 98166, Italy

^2^Infections and Cancer Biology Group, International Agency for Research on Cancer, 150 Cours Albert Thomas, 69372 Lyon, France

^3^Institute of Biomedicine and Translational Medicine, University of Tartu, Estonia

^4^Institute of Translational Pharmacology, CNR, Rome, Italy

*****Corresponding authors: Maria Teresa Sciortino, [mtsciortino@unime.it](mailto:mtsciortino@unime.it); Assunta Venuti, venutia@visitors.iarc.fr.

**Key words**

HSV-1 replication in monocytic THP-1 cell; construction of a recombinant EGFP tagged HSV-1; canonical activation NF-κB mediated by HSV-1; regulation of miRNA-146a by NF-κB HSV-1-mediated; IRAK1 as a target of miR-146a


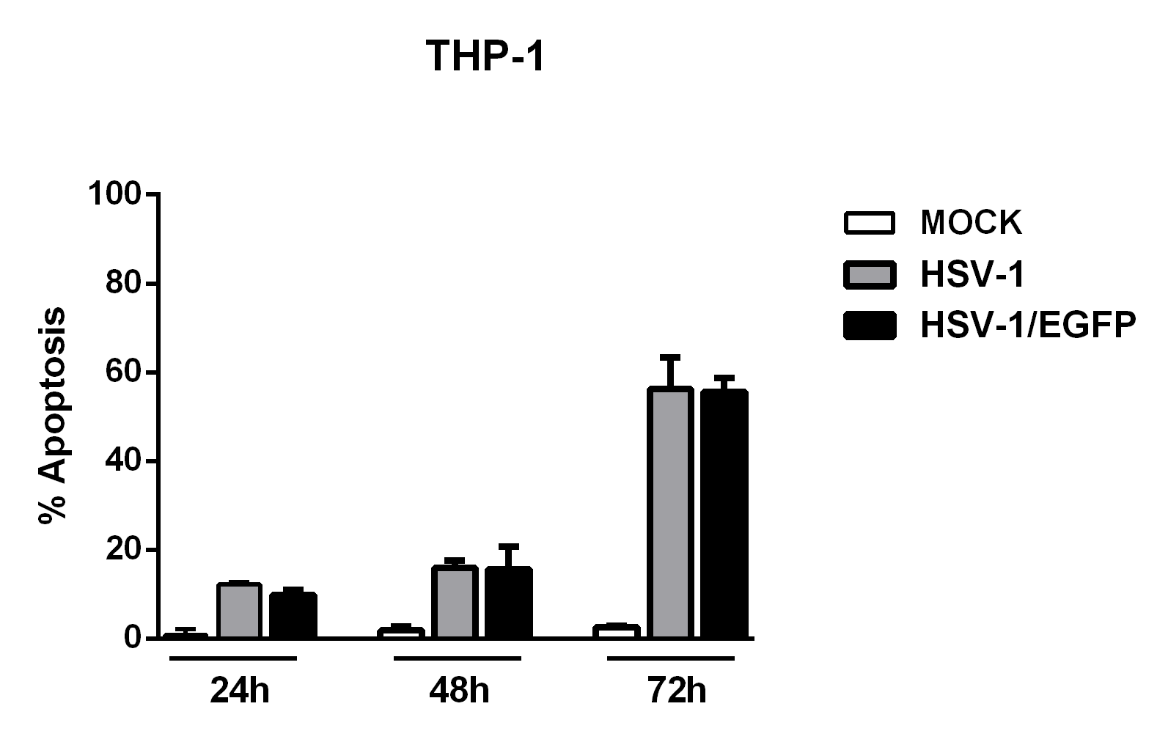


Figure S1: Evaluation of the apoptosis cell death in THP-1, following exposure to the HSV-1. Cells were infected or mock infected at MOI 50 and collected at 24, 48 and 72hrs p.i.. Then, samples were stained with the DNA binding dye acridine orange (SIGMA), showing nuclear morphology of apoptosis by using fluorescence microscopy analysis. The percentage of apoptotic cells was calculated as follows: % apoptosis= Total no. of cells with apoptotic nuclei/Total no. of cells counted X 100.


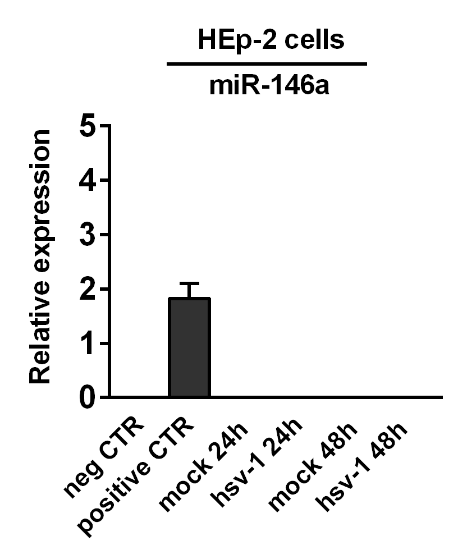


Figure S2: Analysis of miR-146a expression in a permissive HEp-2 cell line after exposure to HSV-1: HEp-2 cells infected or mock infected with HSV-1 at MOI 10, were collected at 24 p.i. and qPCR analysis was performed. THP-1 cells were used as a positive control. Results are the mean ± SD of three independent experiments.


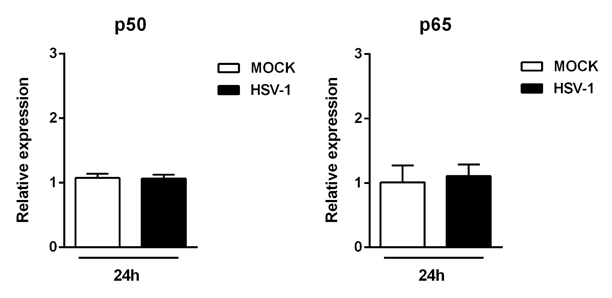


Figure S3: Analysis of p50 and p65 mRNA levels in THP-1 cell after exposure to HSV-1. THP-1 cells were mock infected or infected with HSV-1 at MOI 50 and collected at 24h p.i. qPCR analysis of p50 and p65 was performed by using the following primers: p50-Forw-5’cgtggtgcggctcatgtttac; p50-Rev-5’tttcaagttggatgcattggg; p65-Forw-5’tcagtgagcccatggaattcc; p65-Rev-5’cacagcaatgcgtcgaggtg. Results are the mean ± SD of three independent experiments.
